# LRP/LR specific antibody IgG1-iS18 impedes neurodegeneration in Alzheimer's disease mice

**DOI:** 10.18632/oncotarget.25473

**Published:** 2018-06-05

**Authors:** Eloise Ferreira, Monique J. Bignoux, Tyrone C. Otgaar, Nicolas Tagliatti, Katarina Jovanovic, Boitelo T. Letsolo, Stefan F.T. Weiss

**Affiliations:** ^1^ School of Molecular and Cell Biology, University of the Witwatersrand, Johannesburg, Republic of South Africa; ^2^ Present address: UCL Institute of Ophthalmology, London, UK

**Keywords:** Alzheimer’s disease, amyloid beta, LRP-LR, therapeutic antibody, transgenic mice

## Abstract

Alzheimer’s disease (AD) is a neurodegenerative disease caused by accumulation of amyloid beta (Aβ) plaque and neurofibrillary tangle formation. We have shown *in vitro*, that knock-down and blockade of the 37 kDa/67 kDa Laminin Receptor (LRP/LR) resulted in reduced Aβ induced cytotoxicity and Aβ accumulation. In order to test the effect of blocking LRP/LR on Aβ formation and AD associated symptoms, AD transgenic mice received the anti-LRP/LR specific antibody, IgG1-iS18 through intranasal administration. We show that this treatment resulted in an improvement in memory, and decreased Aβ plaque formation. Moreover, a significant decrease in Aβ_42_ protein expression with a concomitant increase in amyloid precursor protein (APP) and telomerase reverse transcriptase (mTERT) levels was observed. These data recommend IgG1-iS18 as a potentially powerful therapeutic antibody for AD treatment.

## INTRODUCTION

Alzheimer’s disease (AD) is the most prevalent form of neurodegenerative disorders afflicting in excess of 47 million people worldwide [1]. Dementia, including AD poses a significant economic cost, estimated at 818 billion USD in 2015, which is expected to reach 2 trillion USD by 2030 [1]. Due to the considerable knowledge gap in the understanding of the disease causing mechanism, currently only palliative treatment options for AD are available. This highlights the pronounced need to advance the present knowledge base and develop therapeutic strategies for the treatment of AD.

There are two key neuropathological hallmarks of AD; the formation of extracellular amyloid beta (Aβ) plaques and the formation of intracellular neurofibrillary tangles consisting of aggregates of hyper-phosphorylated tau (a microtubule associated protein). Accumulation of these proteins eventually lead to neuronal loss [2]. Aβ plaques are predominantly made up of neurotoxic Aβ_42_ [2] which is generated through the sequential cleavage of the amyloid precursor protein (APP) by β-secretase (also known as BACE1–B-site APP cleaving enzyme) and γ-secretase [3], which results in Aβ shedding. The soluble Aβ oligomers play a role in the formation of ion-permissible channels, therefore causing increased ion influx (of Ca^2+^ in particular) with resultant cytotoxicity [4, 5]. Aβ furthermore induces neuronal loss through an interaction with cell components, either through direct interaction with cell surface receptors [6] or indirectly by incorporating into lipid membranes and cell organelles [7]. One such receptor with which Aβ interacts is the 37 kDa Laminin Receptor Precursor/67 kDa high affinity Laminin Receptor (LRP/LR) [8, 9]. LRP/LR is a multifunctional, non-integrin, type II transmembrane receptor which is predominantly situated within lipid raft regions of plasma membranes as well as within the cytoplasm and the nucleus [10]. LRP/LR serves as a receptor for numerous components including viruses, bacteria, prion proteins, extracellular matrix proteins and very importantly, Aβ. LRP/LR therefore has copious functions in cancer [11, 12], angiogenesis [13], prion disorders [14–17], telomerase [18, 19] as well as AD [6, 8, 20–22]. It was recently discovered that LRP/LR co–localises with APP, indirectly interacts with β- secretase and directly interacts with the catalytic unit (PS1) of γ- secretase to enhance cleavage of APP. In addition, it has been observed that Aβ_42_ interacts with LRP/LR on the cell surface with resultant Aβ_42_ internalisation and accumulation. Treatments targeting LRP/LR, such as the anti-LRP/LR antibody, IgG1-iS18 and shRNAs have shown to result in a significant reduction in Aβ shedding and can furthermore rescue cells from Aβ_42_ induced cytotoxicity [8, 21, 23].

Another such protein with which LRP/LR interacts is the reverse transcriptase ribonucleoprotein, telomerase. Telomerase predominantly plays a role in maintaining telomeres in highly proliferative cells [24, 25] by catalysing the addition of TTAGGG repeats to telomeric DNA to protect telomeres from erosion. Telomerase consists of two essential components, the TERT enzyme which is responsible for the reverse transcriptase activity and the telomerase RNA template component, TERC [26]. Telomerase has furthermore been implicated in the pathological process of AD. It has been shown that people suffering from AD have shorter telomere lengths in their neuronal and T cells [27] and that Aβ_42_ inhibits telomerase activity through the binding of Aβ oligomers to the telomeric DNA-RNA template complex of telomerase [28]. This indicates an antagonistic relationship between telomerase and Aβ within neurons. We have recently discovered that LRP/LR and hTERT interact, both on the cell surface and within the perinuclear compartments [19, 23]. Together with this, it was observed that knockdown of LRP/LR mediated by small interfering RNAs (siRNAs) caused a significant impediment of telomerase activity, indicating that LRP/LR has a function in regulating telomerase activity [18, 23].

TERT furthermore performs extra-telomeric functions, whereby it is known to play a protective role in the mitochondria against apoptosis, mitochondrial DNA damage [29] and is involved in DNA damage responses and repair [30]. It has been observed that DNA damage is an initial and critical contributor for aging and is affected by the accumulation of persistent DNA lesions containing unrepairable double strand DNA breaks (DSBs) [31]. Oxidative damage caused by free radical/ROS formation can lead to formation of DSBs which can trigger a series of repair pathways. These involve replacement of histone H2A with the histone variant H2AX, in nucleosomes which flank DSBs with concurrent or subsequent phosphorylation of a C-terminal serine in H2AX. Phosphorylated H2AX (γH2AX) helps recruit proteins containing phospho-specific interaction domains which in turn help recruit DNA repair factors [32]. Accumulating evidence suggests increased DNA damage, particularly oxidative damage, and deficiencies in the repair of DNA lesions in cells from patients with AD [33]. Importantly, Aβ peptides directly initiate free radical/ROS formation, cellular dysfunction, and subsequent neuronal death [31]. Hence, protection from DNA damage presents a basic approach for treatment of age-related diseases such as AD.

With this information in mind, we aimed to investigate LRP/LR as a potential therapeutic target for the treatment of AD in 5XFAD mice. In order to examine this, LRP/LR was blocked with IgG1-iS18 antibody and the effect of the treatment on AD related brain pathology, memory and the cognitive abilities of the mice were studied. In addition, levels of Aβ and other AD related proteins, γH2AX as well as TERT expression and telomerase activity were determined.

## RESULTS

### IgG1-iS18 significantly improves short term memory and learning ability of 5XFAD mice

**Figure 1:**
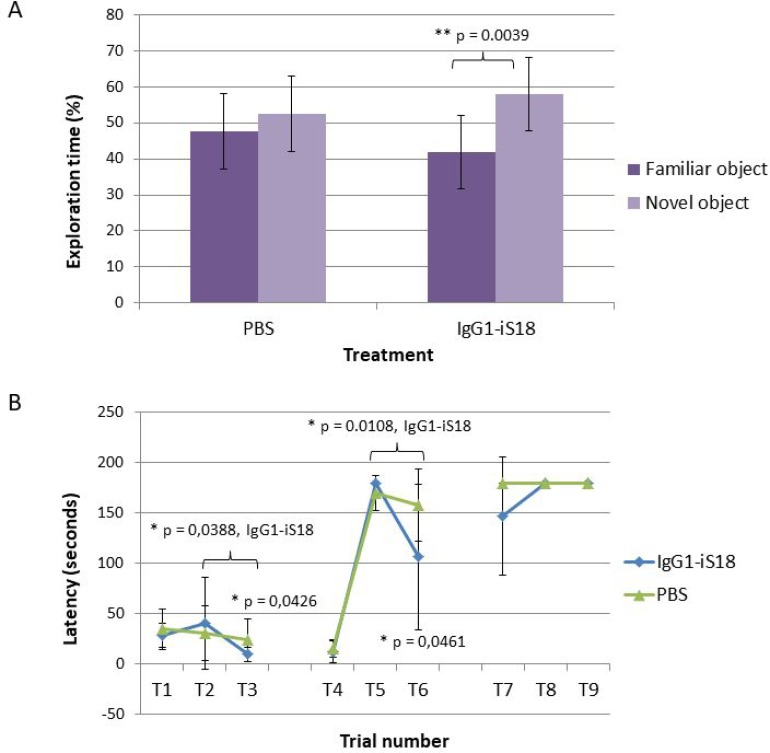
Memory and cognitive function tests performed to assess the effect of treatment with IgG1-iS18 (**A**) The novel object recognition test was performed with 4–5 month old 5XFAD (*n* = 10) male mice treated with either PBS or IgG1-iS18. The percentage of time that the mice spent exploring the novel and familiar object over the total time spent exploring both objects was calculated. Error bars represent standard deviation, ^*^p, 0.05, ^**^p, 0.01, ^***^p, 0.001; One-way ANOVA. (**B**) Performance of the AD transgenic mice in the puzzle box test. Latencies scored to reach the goal zone during the 9 trials of the test are shown. Tasks performed during T1–T9 are as follow: Day 1 (training), T1 - underpass un-blocked, and the top of the underpass uncovered, T2 and T3 the top was covered and mice entered the goal box via the underpass. Day 2 (burrowing puzzle), T4 was identical to T2 and T3. T5 and T6 - underpass was filled with sawdust. Day 3 (plug puzzle), T7 was a repetition of T5 and T6. T8 and T9 underpass was obstructed by a plastic object. Error bars represent standard deviation, *n* = 12 per group, ^*^p, 0.05, ^**^p, 0.01, ^***^p, 0.001; One-way ANOVA.

In order to validate whether targeting LRP/LR using IgG1-iS18, in AD transgenic mice would have an effect on their cognitive abilities, the novel object recognition and puzzle box tests were performed. We carried out a intranasal treatment protocol twice a week for 8 weeks in 5XFAD transgenic mice beginning at the age of approximately 4–5 months. Mice received either PBS as control treatment or the anti-LRP/LR specific antibody, IgG1-iS18. After completion of the treatment protocol the novel object recognition test was performed and the percentage time exploring a familiar as well as novel object was recorded. A one-way between subjects ANOVA was conducted to compare the effect of treatment on the time exploring familiar and novel objects. The PBS treated mice showed no significant difference in the time spent exploring the familiar and novel objects [F(1, 18) = 1.09, *p* = 0.311]. However, mice that received IgG1-iS18 via intranasal administration spent 16.18% more time exploring the novel objects [F(1, 16) = 11.36, *p* = 0.0039] (Figure 1A). Post hoc analysis using the Tukey-HSD test indicated that the mean value for the exploration time (percentage) of the novel object (Mean = 58.09%, SD = 10.18%) was significantly different than the percentage time exploring the familiar objects (Mean = 41.91%, SD = 10.18%). Next, the puzzle box test was performed to determine the effect of intranasal administration on short and long term memory as well as the learning ability of the 5XFAD mice (Figure 1B). When analysing short term memory, the PBS treated mice were significantly impaired on T3 and T6, and had higher latencies to reach the goal zone compared to the mice that received IgG1-iS18 (T3 [F(1, 20) = 4.69, *p* = 0.0426] and T6 [F(1, 21) = 4.49, *p* = 0.0461]. Post hoc analysis further showed that the IgG1-iS18 treated mice (T3: Mean = 9.34 s, SD = 6.61 s; T6: Mean = 106.21 s, SD = 72.75 s) reached the goal zones in trial T3 and T6 in significantly less time than the PBS treated mice (T3: Mean = 23.77 s, SD = 21.09 s; T6: Mean = 157.82 s, SD = 36.39 s). One-way ANOVA and Tukey-Kramer post hoc analysis furthermore showed that the IgG1-iS18 treated group displayed learning of the open as well as the burrow puzzle with a significant decrease in latencies from 40.2 s (T2) to 9.3 s (T3) (T2 vs. T3, *p* = 0.0388) and 180 s (T5) to 106.2 s (T6) (T5 vs. T6, *p* = 0.0108) respectively. In addition, PBS treated mice had higher latencies to reach the goal zone on T7, when analysing long term memory, though not significantly when compared to IgG1-iS18 treated mice (Mean (PBS) = 180, SD = 0; Mean (IgG1-iS18) = 146.73, SD = 58.73; *p* = 0.06) (Figure 1B).

### Intranasal administration of IgG1-iS18 decreases AD histopathological hallmarks and Aβ levels in brains of AD transgenic mice

**Figure 2:**
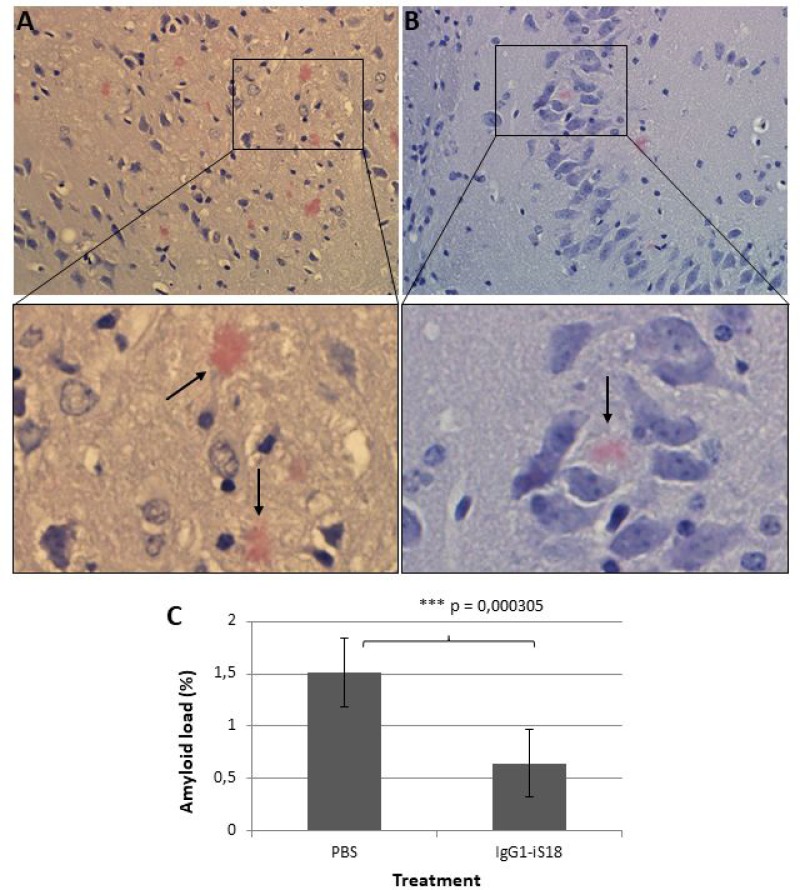
Congo red stain of the hippocampus of AD transgenic mice treated with (**A**) PBS and (**B**) IgG1-iS18. Amyloid load (**C**) is expressed as the proportion (%) of tissue area occupied by amyloid beta plaques in the hippocampal sections of the PBS and IgG1-iS18 treated mice. A decrease in amyloid plaque formation is observed after treatment with IgG1-iS18. Original magnification at 400×. Error bars represent standard deviation, *n* = 7 per group, ^*^p, 0.05, ^**^p, 0.01, ^***^p, 0.001; One-way ANOVA.

**Figure 3:**
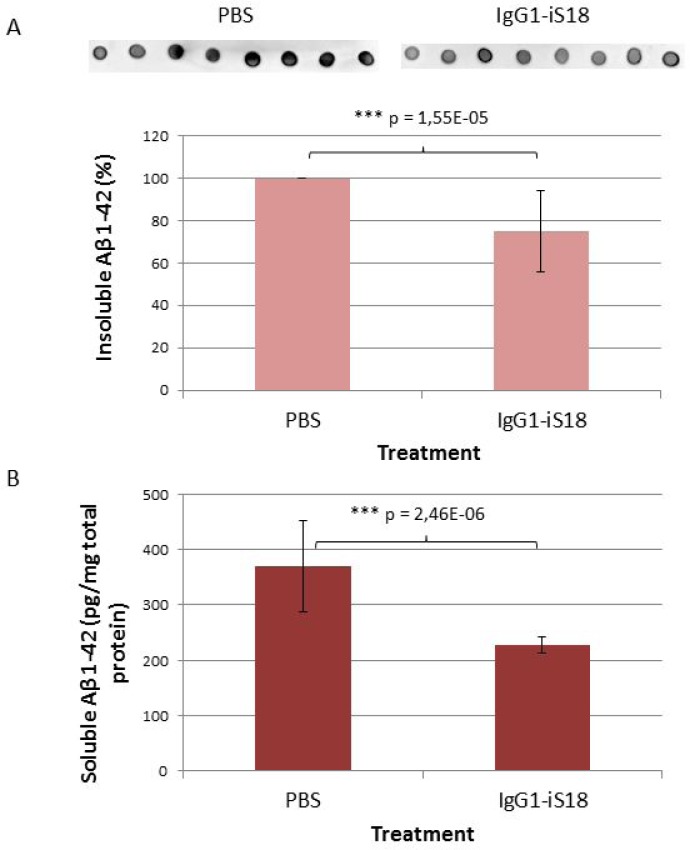
Levels of Aβ in brain tissue of AD transgenic mice after treatment with IgG1-iS18 and PBS (**A**) Insoluble Aβ levels were determined by dot blot and calculated as a percentage per mg brain tissue. Data shown (average ± standard deviation) is representative of eight biological repeats (performed in duplicate) per treatment group. ^*^p, 0.05, ^**^p, 0.01, ^***^p, 0.001; One-way ANOVA. (**B**) Levels of soluble Aβ in brain tissue of AD transgenic mice after treatment with IgG1-iS18 and PBS as determined by Aβ_42_ ELISA. Data shown (average ± standard deviation) was calculated as pg Aβ_42_ per mg total protein and is representative of seven biological repeats per group (seven mice per treatment group, performed in duplicate). ^*^p, 0.05, ^**^p, 0.01, ^***^p, 0.001; One-way ANOVA.

After sacrifice at 9 months, histological studies were performed utilizing the Congo red stain. The visual observation of these images revealed that treatment with IgG1-iS18 antibody (Figure 2B) caused a decrease in amyloid plaques in the hippocampus when compared to mice that received PBS (Figure 2A) treatment. Quantitative studies and post hoc analysis of the amyloid load (%) in the hippocampal sections of the PBS and IgG1-iS18 treated mice showed that there was a significant decrease after treatment with IgG1-iS18 (Mean = 0.64%, SD = 0.32%) when compared to the PBS treated mice (Mean = 1.52%, SD = 0.33%) [F(1, 12) = 25.08, *p* = 0.00031] (Figure 2C). To confirm the effect of intranasal IgG1-iS18 antibody treatment on amyloid levels, insoluble and soluble Aβ levels of the entire contralateral hemisphere of the mouse brains, from each of the two treatment groups were determined. Insoluble protein was extracted with guanidine hydrochloride (GuHCl) and dot-blot analysis was performed to determine the levels of Aβ per mg brain tissue. It was observed that there was a significant 24.72% [F(1,30) = 26.46, *p* = 1.55E-05] decrease in insoluble Aβ_1-42_ after treatment with IgG1-iS18 (Mean = 75.28%, SD = 19.22%) when compared to the PBS control mice (Mean set to 100%) (Figure 3A). Next, we investigated the level of soluble Aβ_1-42_ peptide by performing sandwich ELISAs. A highly significant decrease [F(1,24) = 37.61, *p* = 2.46E-06] was observed (Figure 3B) when the average Aβ_1-42_ level of the nasal IgG1-iS18 antibody treated mice (Mean = 227.71 pg/mg, SD = 15.06 pg/mg) was compared with that of the PBS treated mice (Mean = 370.21 pg/mg, SD = 82.41 pg/mg).

Together, these data suggest that there is a significant decrease in Aβ protein levels in the mouse brain homogenates after treatment with IgG1-iS18.

### IgG1-iS18 treatment substantially increases APP brain levels

**Figure 4:**
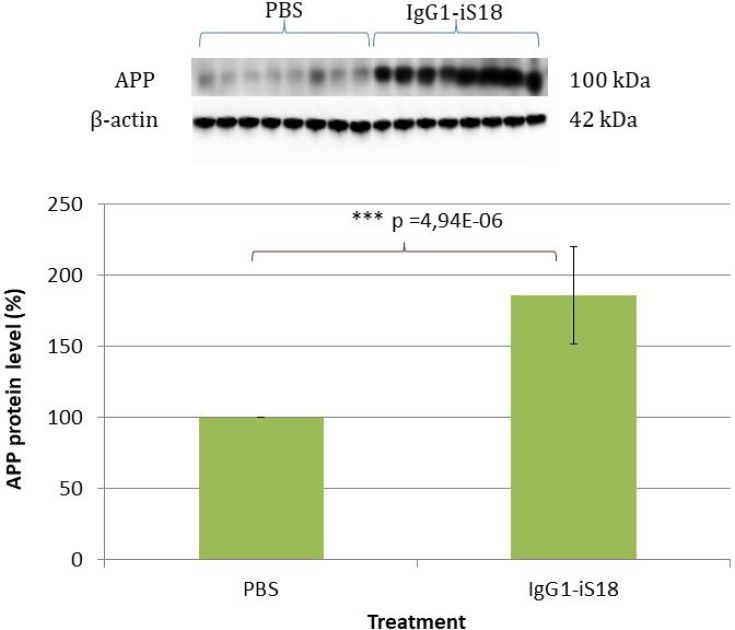
Western blot analysis of APP levels in brain tissue of AD transgenic mice after treatment with IgG1-iS18 and PBS A significant increase in APP levels was observed after treatment with IgG1-iS18. Error bars represent standard deviation, *n* = 8 mice per treatment group. ^*^p, 0.05, ^**^p, 0.01, ^***^p, 0.001; One-way ANOVA.

Since we have previously reported that IgG1-iS18 treatment had no effect on cell surface levels of APP, β-secretase and γ-secretase in HEK293 cells, *in vitro*, [20] we wanted to confirm that levels of these proteins in the brain were the same in all treatment groups. Western blotting revealed uniform expression of LRP, β- and γ-secretase among the PBS and antibody treated animals (Supplementary Figure 1). This was expected as IgG1-iS18 blocks the interaction between LRP and β-, and γ-secretase, respectively, and therefore, does not affect protein levels [21, 22]. However, in contrast to these previously published observations, APP levels increased substantially and significantly after antibody treatment of the 5XFAD transgenic mice, with an average elevation of 85.86% [F(1,14) = 51,11, *p* = 4.94E-06] observed (Figure 4).

### Intranasal IgG1-iS18 treatment significantly increases mTERT expression and phosphorylation of H2AX

**Figure 5:**
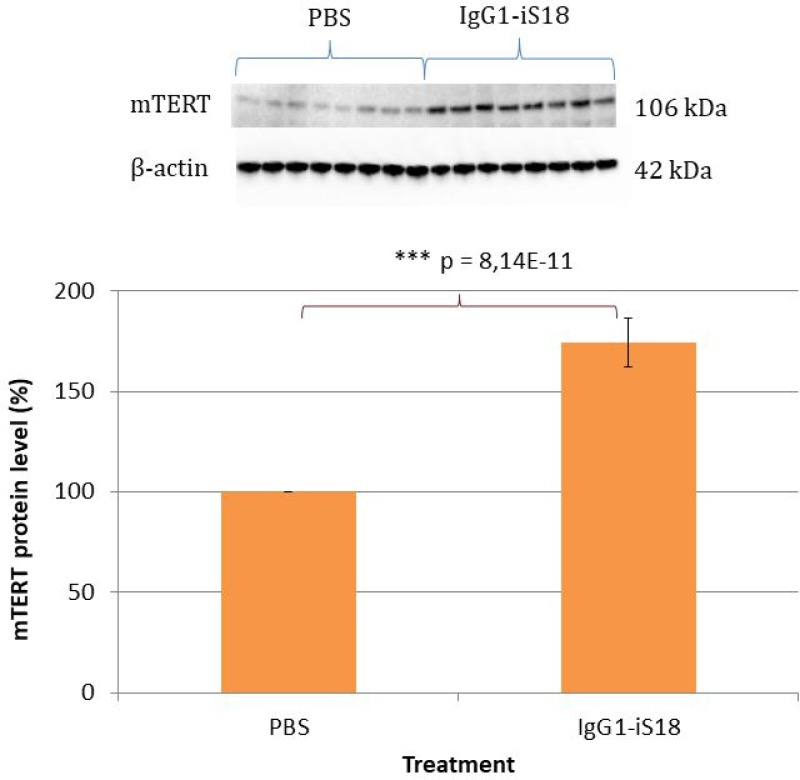
Levels of mTERT in brain tissue of AD transgenic mice after treatment with IgG1-iS18 and PBS Western blot analysis was performed and a significant increase in mTERT levels was observed after treatment with IgG1-iS18. Error bars represent standard deviation, *n* = 8 mice per treatment group, ^*^p, 0.05, ^**^p, 0.01, ^***^p, 0.001; One-way ANOVA.

We have recently shown that LRP/LR co-localizes with the reverse transcriptase ribonucleoprotein, TERT [18]. It has furthermore been reported that TERT plays a neuroprotective role [34] and that there is a reduction in expression of TERT protein in the brain, during normal aging in mice [35]. Therefore, we decided to investigate mTERT protein expression in the mouse hemi-brain homogenates. Western blot analysis revealed an extensive and highly significant 74.34% [F(1,14) = 296.29, *p* = 8.14E-11] increase in mTERT protein levels in the brain tissue of the IgG1-iS18 antibody treated mice compared to the PBS control group (Figure 5). Furthermore, post hoc analysis using the Tukey HSD test confirmed this observation. Moreover, since TERT is responsible for the reverse transcriptase activity of telomerase, we determined the effect of the IgG1-iS18 antibody treatment on telomerase activity in the mouse brain tissue. We detected extremely low levels of telomerase activity in the mouse brain homogenates of both the PBS as well as IgG1-iS18 treated mice and no significant change between control and treated mice was observed (Supplementary Figure 2). This is in agreement with previous findings describing downregulation of telomerase activity in rodent brain postnatally [36].

**Figure 6:**
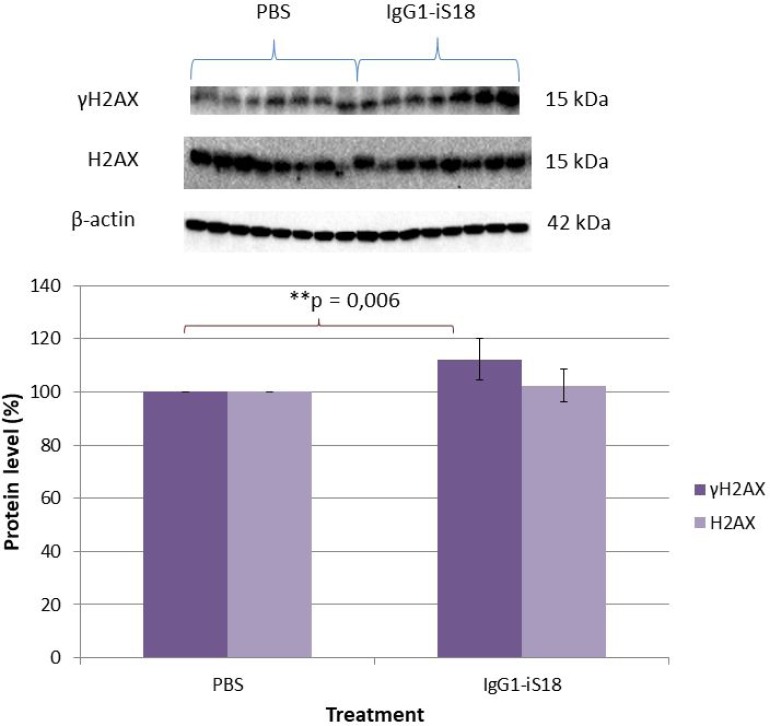
Effect of IgG1-iS18 on phosphorylated (Ser139) γH2AX and total H2AX protein levels in brain tissue of AD transgenic mice as detected by western blot analysis A significant increase in pSer139 γH2AX levels were observed after treatment with IgG1-iS18. Error bars represent standard deviation, *n* = 7 mice per treatment group. ^*^p, 0.05, ^**^p, 0.01, ^***^p, 0.001; One-way ANOVA.

It has been established that DNA damage is an initial and critical contributor for aging and it has been suggested that the pathogenesis of AD may involve a DNA repair defect [33]. To further substantiate the neuroprotective role of IgG1-iS18 and to investigate the effect of this treatment on the DNA damage response, the levels of phosphorylated H2AX (γH2AX) was determined. These histones are specifically phosphorylated at Ser139 and serve to mark sites of DNA damage and help recruit DNA repair factors [32]. We performed western blot analysis with protein extracted from hemi-brains of mice that received either PBS or IgG1-iS18 antibody treatment to determine the levels of γH2AX. Figure 6 shows there was a significant increase [F(1,12) = 11.34, *p* = 0.00559] in levels of γH2AX but not in endogenous levels of total H2AX [F(1,12) = 1.06, *p* = 0.3246], in the brain tissue of antibody treated mice when compared to PBS treated control mice.

## DISCUSSION

Here we demonstrate the novel finding that intranasal administration of the anti-LRP/LR specific antibody, IgG1-iS18 to 5XFAD transgenic mice results in improved recognition and learning/short term memory as well as decreased Aβ accumulation with a concomitant increase in mTERT levels. Since we have previously observed *in vitro*, that blockade of LRP/LR with IgG1-iS18 reduced Aβ shedding, hampered Aβ_42_ internalization and increased cell survival in the presence of Aβ, we hypothesized that intranasal administration of IgG1-iS18 might lead to reduced Aβ generation and toxicity and alleviate AD associated symptoms. In testing this hypothesis, we found that biweekly nasal administration of IgG1-iS18 for 8 weeks significantly improved recognition and short term memory (Figure 1).

Cognitive impairments in AD are exhibited as deficits in higher-order neurocognitive functions including; planning and problem solving, short term, long term and working memory. There are various potential memory and cognitive function assessments routinely used to investigate cognitive decline in neurodegenerative diseases. The object recognition test is now among the most commonly used behavioural tests for mice. This test assesses attention to novelty and exploration of a novel, non-aversive object placed in a familiar environment [37, 38]. The novel object recognition test was therefore used to evaluate the effect of treatment with IgG1-iS18 on recognition memory. We observed that the treated mice exhibited object recognition by preferentially exploring the novel object (58.09%) compared to the familiar object (41.91%) during the testing phase as indicated in Figure 1A. In a study performed by Frydman-Marom *et al.*, [39] they observed that wild type mice that do not express the 5XFAD transgene spent approximately 68% of the total exploration time exploring the novel object. This suggests that treatment of 4–5 month old 5XFAD mice with IgG1-iS18 can improve object recognition but not to baseline levels as observed in wild type mice. It is however possible that initiating treatment at an earlier stage and increasing the number of treatments might decrease Aβ levels even further and improve the memory and cognitive abilities of the AD transgenic mice. Nevertheless, a significant improvement was observed in recognition memory in the 5XFAD transgenic mouse model that shows rapid synthesis of Aβ, neuronal loss and neuroinflammation [40].

To further investigate the effect on short and long term memory as well as learning ability of the AD transgenic mice, the puzzle box test was performed. This is a problem-solving test in which mice are required to complete escape tasks of increasing difficulty within a limited amount of time. It has previously been shown to be a quick but highly reliable assessment of higher-order cognitive functioning [41]. We determined that the PBS treated control mice had higher latencies to reach the goal zone on T3 and T6, when the underpass was open and blocked with sawdust (burrow puzzle), respectively when compared to mice treated with IgG1-iS18. IgG1-iS18 treated mice furthermore showed a significant decrease in latencies to reach the goal zone in T3 and T6 when compared to T2 (underpass open) and T5 (underpass blocked with sawdust) respectively, whereas, no difference was observed for the PBS treated control mice (Figure 1B) This suggests an improvement in learning/short term memory on the immediate problem repeats trials. Girard *et al.*, [42] showed that deficits in learning and memory in 5XFAD transgenic mice started at 4 months with a substantial increase at 6 months of age. This further suggests that treatment should be initiated prior to the onset of cognitive impairment which might explain why no significant improvement was observed in the long term memory or problem solving abilities of the 4–5 month old 5XFAD mice treated with IgG1-iS18 in the current study. Nonetheless, we still observed a significant improvement in recognition and learning/short term memory after intranasal treatment with IgG1-iS18. In light of the improvement in memory after treatment with IgG1-iS18, we decided to investigate the effect on the histopathology of the hippocampus of the AD mice and we observed a marked decrease in amyloid plaque formation (Figure 2). It has previously been ascertained that hippocampal lesions cause moderate memory impairment [43] and altered executive functions in the puzzle box test [41]. We, therefore, suggest that the improvement in memory and cognitive function is due to the improvement in AD brain pathology.

To confirm if the nasal IgG1-iS18 treatment had an effect on Aβ levels, we performed ELISA and dot-blot quantification of Tris-soluble (soluble) and GuHCl-soluble (insoluble) Aβ_42_ peptide levels respectively, in mouse hemi-brain homogenates. We found a significant decrease in the average Aβ burden, selectively in the IgG1-iS18 treatment group (Figure 3). This is in agreement with our previously published observations, whereby, treatment of HEK293 and SH-SY5Y cells with IgG1-iS18 caused a decrease in Aβ levels [20]. In addition, we have previously demonstrated that LRP/LR and Aβ_42_ interact on the cell surface and that the LRP/LR—Aβ_42_ association results in the induction of apoptosis, which may be significantly deterred upon blockade of this association with anti-LRP/LR-specific antibodies. This study further demonstrated that LRP/LR plays a central role in mediating Aβ_42_ internalization and that antibody blockade and shRNA-mediated down-regulation of the receptor significantly impedes cellular Aβ_42_ uptake and consequent apoptosis [44]. Therefore, it is likely that the responses in cognitive ability and brain pathology observed selectively in the IgG1-iS18 antibody treated animals are causally related to the significantly lower brain Aβ levels seen only in these mice. Moreover, we suggest a possible decrease in Aβ_42_ internalization and therefore a subsequent decrease in neurotoxicity.

We have previously shown, by FRET analysis [21], that LRP/LR directly interacts with γ-secretase and indirectly interacts with β-secretase and that blockade of LRP/LR by IgG1-iS18 impedes these interactions and consequent Aβ_1-42_ shedding [22]. This, therefore, suggests that treatment with this antibody does not affect the levels of these AD related proteins but only prevents the sequential cleavage of APP by β- and γ-secretase to prevent formation and shedding of Aβ_1-42_. In light of these previously obtained *in vitro* results, we wanted to confirm that IgG1-iS18 antibody treatment had no significant effect on the levels of LRP/LR, β- and γ-secretase as well as APP in the mouse brain tissue when compared to the PBS treated control mice. Western blotting revealed uniform LRP/LR, β- and γ-secretase expression among the animals (Supplementary Figure 1). However, an interesting finding, in contrast to what we previously observed *in vitro*, was that treatment with the anti-LRP/LR antibody caused a significant 85.86% increase in APP levels *in vivo* (Figure 4). We, therefore, suggest that the increase in APP levels is due to reduced cleavage of this protein, owing to the diminished interaction between LRP/LR, β- and γ-secretase [22], which is concurrent with the reduction in Aβ_1-42_ production that we observed (Figure 3).

In addition to the interactions we have observed between LRP/LR, β- and γ-secretase [22], we have recently shown that LRP/LR and the reverse transcriptase ribonucleoprotein, TERT, co-localizes in tumorigenic as well as non-tumorigenic cells and co-immunoprecipitation assays furthermore confirmed an interaction between these proteins [18]. In rodent brain, telomerase activity is high during embryonic development but rapidly decreases postnatally [36]. However, expression of TERT has been described to persist into adulthood in mouse and rat [36]. In agreement with this, we observed very low levels of telomerase activity in the mouse brain homogenates with no significant change between control and treated mice (Supplementary Figure 2). Besides the role TERT plays as reverse transcriptase ribonucleoprotein in telomerase, TERT also performs extra-telomeric functions. It has been reported that TERT protects neurons from apoptosis induced by various stresses [34] and moreover, is involved in DNA damage responses and repair [30]. Considering the known protective functions of TERT, we hypothesized that an increase in mTERT- levels could confer resistance to amyloid pathology. We, therefore, wanted to determine if the improvement in cognitive ability and decrease in Aβ observed after antibody treatment was accompanied by a concomitant increase in mTERT levels. Interestingly, we discovered a significant increase in mTERT protein levels in the brain tissue of the antibody treated mice when compared to the PBS treated control animals (Figure 5). It is well-known that the incidence rate of AD increases with age and it has recently been shown that the pathological mechanisms of AD are associated with human telomerase. Wang *et al.*, [28] found that Aβ_1-40_ and Aβ_1-42_ inhibit telomerase activity *in vitro* by binding to the telomeric DNA/RNA complex of telomerase. In addition, Zhu *et al.*, [45] demonstrated a neuroprotective function of TERT in an *in vitro* AD experimental model, whereby overexpression of TERT decreased vulnerability to Aβ-induced apoptosis. Therefore, we suggest that in the current study, the blockade of the LRP/LR—Aβ_42_ association by IgG1-iS18 prevented internalization of extracellular Aβ_42_ and subsequent apoptosis. Moreover, the decrease in Aβ levels observed after IgG1-iS18 antibody treatment caused a reduction in neurotoxicity and hence a concomitant increase in mTERT levels. It is also possible that the increase in mTERT levels has a protective function in the brain and may offer further neuronal resistance against pathological Aβ.

It has been suggested that TERT likewise plays a role in resistance to tau pathology. Spilsbury *et al.*, [46] recently observed, *in vitro,* that in the absence of TERT, pathological tau increases ROS generation and oxidative damage in neurons. They furthermore observed a mutual exclusion of TERT and neurofibrillary tangles (NFT) in the hippocampus of AD patients, suggesting that TERT expression might further play a role in tau pathology. However, Maarouf *et al.*, [40] observed no elevations in Tris-soluble or GHCl-soluble mouse endogenous tau in 5XFAD transgenic mice with increasing age. No NFT or phosphorylated-tau staining was observed in 5XFAD mouse brain sections using the AT8 antibody. Furthermore they suggested an inability of high Aβ loads to cause NFT in 5XFAD mice [40] and therefore the effect of IgG1-iS18 treatment on NFT and phosphorylated-tau levels was not investigated in the current study.

As previously mentioned, studies have elucidated that Aβ neurotoxicity is associated with Aβ induced neuronal apoptosis via oxidative stress and disrupted cellular calcium homeostasis, which both contribute to DNA damage [47, 48] and mitochondrial dysfunction [49]. Several factors involved in DNA repair and in signalling the presence of damage have shown to accumulate after double-strand DNA breaks. One such important response to DNA damage is the phosphorylation of H2AX (γH2AX) [50] which facilitate the recruitment of a subset of damage response and repair proteins. Masutomi *et al.*, [51] showed that in cells lacking hTERT, the DNA damage response was impaired due to a reduced degree of phosphorylation of H2AX, thus implicating hTERT as a critical regulator of the DNA damage response pathway. Since we observed a decrease in neurotoxicity and an increase in mTERT levels after treatment with IgG1-iS18, we decided to investigate the levels of γH2AX present in the brain tissue. Surprisingly, the antibody treatment significantly increased levels of γH2AX but not total H2AX (Figure 6). This suggests that the increase in γH2AX levels observed was as a result of phosphorylation of H2AX rather than an increase in total endogenous H2AX levels. It is possible that the increase in the γH2AX levels was due to the concomitant increase in mTERT and might contribute to the repair and protection against neurodegeneration as observed in this study due to the involvement of γH2AX in the recruitment of a multitude of DNA damage response proteins.

In summary, this is the first report proposing that intranasal administration of the anti-LRP/LR antibody, IgG1-iS18, is able to reduce levels of soluble and insoluble Aβ_42_ in the whole brain and diminish accumulation of amyloid plaques in the hippocampus. We suggest that, together with this, the increase in mTERT levels and H2AX phosphorylation resulted in a decrease in neurotoxicity and consequently conveyed improvement of cognitive abilities, recognition and learning/short term memory. Therefore, we recommend the anti-LRP/LR specific antibody, IgG1-iS18, as a novel and powerful potential therapeutic strategy for treatment of AD. This therefore prompts the implementation of clinical studies to further investigate the effect of IgG1-iS18 on patients suffering from AD.

## MATERIALS AND METHODS

### Animals and intranasal treatment

The B6SJL-Tg(APPSwFlLon, PSEN1^*^M146L^*^L 286V)6799Vas/Mm (Tg6799) transgenic line was used and obtained from Jackson Laboratories (Bar Harbour, ME). These 5XFAD transgenic mice overexpress mutant human APP(695) with the Swedish (K670N, M671L), Florida (I716V), and London (V717I) Familial Alzheimer’s Disease (FAD) mutations along with human PS1 harbouring two FAD mutations, M146L and L286V. Both transgenes are regulated by the mouse Thy1 promoter to drive overexpression in the brain. 5XFAD mice display major features of Alzheimer’s disease amyloid pathology and are useful models of intraneuronal Aβ_42_ induced neurodegeneration and amyloid plaque formation. The high APP expression observed in this model causes accumulation of Aβ_42_, and plaque formation can be observed at 2 months of age. Twenty four male mice (at approximately 4.5 months) were randomized to 2 treatment groups: group 1 (*n* = 12) received PBS as vehicle control and group 2 (*n* = 12) received the anti-LRP specific antibody IgG1-iS18. The treatments were administered intranasally twice a week for 8 weeks and the mice received IgG1-iS18 at a total dose of 288 μg/ mouse (9 μg/15 μl/naris = 18 μg/mouse, 2 × week = 36 μg/mouse/week, 8 weeks = 288 μg/ mouse). All procedures were performed with approval by the University of the Witwatersrand Animal Ethics Screening Committee (clearance certificate number 2014/37/C).

### Memory and cognitive function tests

#### Novel object recognition

The novel object recognition test *(for reviews see* [52, 53]) was performed in a 440 mm × 500 mm open field chamber with opaque walls. Each mouse was habituated to an empty novel object recognition open field box for two 10 min sessions 24 hr apart. Twenty four hours after the last habituation session, mice were subjected to training in a 10 minute session of exposure to two identical, non-toxic, hard plastic items in the open field box. After the training session, the animal was returned to its home cage. After a retention interval of 24 hrs, the animal was returned to the arena which contained two objects, one identical to the familiar object and one novel object. The animal was allowed to explore for 10 minutes, during which the amount of time exploring each object was recorded and tracked employing Panlab Smart video tracking software, Smart 3.0. Objects were randomized and counterbalanced across animals. Objects and arenas were thoroughly cleaned between trials.

#### Puzzle box test

The protocol was slightly modified from Ben Abdallah *et al.*, [41]. The arena consisted of a white box divided by a removable barrier into two compartments: a start zone (67 cm long, 25.5 cm wide) and a smaller dark and covered goal zone (15 cm long, 25.5 cm wide). The mice were introduced into the start zone and allowed to move into the goal zone through a narrow underpass (∼4 cm wide) located in the barrier. The mice were subjected to a total of nine trials (T1–T9) over 3 consecutive days, with three trials per day, during which they were challenged with obstructions of increasing difficulty placed at the underpass. On day 1 (training), the underpass was un-blocked, and the top of the underpass was uncovered during T1. During T2 and T3 the top was covered and mice entered via the small underpass. On day 2 (burrowing puzzle), T4 was identical to T2 and T3. During T5 and T6, however, the underpass was filled with sawdust and mice had to dig their way through. On day 3 (plug puzzle), T7 was a repetition of T5 and T6. However, during T8 and T9, mice were presented with the plug puzzle, where the underpass was obstructed by a plastic object that mice had to pull or push with teeth and paws to enter the goal zone. This sequence allowed assessing problem solving ability (T5 and T8), learning and short-term memory (T3, T6, and T9) while the repetition on the next day provided a measure of long-term memory (T4 and T7). Performance of mice in the puzzle box was assessed by measuring the latency to enter the goal zone with all four paws or was ended after a total time of 3 min.

### Brain harvest

After all memory and cognitive tests were performed mice were euthanized and transcardially perfused with cold phosphate buffered saline (PBS) (Sigma-Aldrich). A hemibrain from each mouse was dissected, snap-frozen in liquid nitrogen, then stored at –70° C until used for biochemical analysis. The remaining hemibrains were fixed in 4% paraformaldehyde (Sigma-Aldrich) for histological analysis. Briefly, the brains were fixed for 24 h in 4% paraformaldehyde at 4° C where after they were rinsed with PBS. In order to displace all water from the brain samples, 30% sucrose was added and the samples were incubated for approximately 3 days. The samples were then stored at 4° C in 0.1M PBS containing protease inhibitor (Sigma-Aldrich) until further analysis.

### Histological analysis

Histological analysis was performed by IDEXX laboratories (Pretoria, South Africa). All brain samples (*n* = 8) were re-fixed in 10% buffered formalin and sections were made and processed according to routine histological tissue processing in an automated tissue processor with standard operating procedures Idexx-AP-SOP-27. Following tissue processing, sections of 5–6 μm were cut (IdexxSA-AP-SOP-30) and the produced slides were stained in an automated Haematoxylin and Eosin tissue stainer (IdexxSA-AP-SOP-205). Slides were furthermore stained with Congo red to identify amyloid before histological evaluation. The amyloid load was quantified from histological sections by using ImageJ software. Amyloid plaques were manually outlined and the amyloid load corresponded to the ratio (%) of the mean area of amyloid plaques to total area measured in each hippocampal section.

### Protein extraction

All steps were completed at 4° C. The frozen brain samples were homogenized in 500 μl cold PBS with protease inhibitors using a Dounce homogenizer. Of each sample, 100 μl homogenate was removed and frozen at –20° C for analysis of telomerase activity. To the remaining homogenate, 400 μl of 2× RIPA buffer was added and the samples were lysed at 4° C for 30 min with gentle agitation. The supernatant, containing the soluble proteins, was collected after centrifugation at 16000 × g for 20 min and stored at –20° C until further analysis. The remaining tissue pellet was then homogenized in 100 μl Guanidine HCl buffer (GuHCl) (5M Guanidine HCl, 50 mM Tris (pH8) containing protease inhibitors (Sigma-Aldrich)) to extract insoluble amyloid beta. After homogenization, 7.9 ml GuHCl was added and the samples were gently rotated overnight at 4° C. The supernatant was stored at –20° C until further analysis. A BCA assay was performed on the soluble protein samples in order to determine the total protein concentration.

### Western blot analysis

The soluble protein samples were heated for 5 min at 95° C in Blue Loading Buffer (New England Biolabs) with 40 mM of dithiothreitol and a total of 10 μg (β-actin), 20 μg (γH2AX), 25 μg (PS1), 35 μg (LRP), 40 μg (APP and H2AX), 50 μg (mTERT) and 60 μg (BACE1) of protein was then separated on AnykD™ Criterion™ TGX Stain-Free™ Protein Gels (Biorad). A prestained molecular weight marker (PageRuler Prestained Protein Ladder, Thermo Fisher) was loaded onto each gel. After transferring the proteins onto polyvinylidine fluoride membrane (Pall) with 1 × transfer buffer (20% methanol in 25 mM Tris and 19.2 mM glycine) the membranes were blocked in 3% BSA (Amresco) in PBS and 0.1% Tween 20 (PBST). The primary and secondary antibodies used for western blots are detailed in Supplementary Table 1. The proteins were visualized with Clarity™ Western ECL Blotting Substrate (Biorad) and the ChemiDoc™ Imaging System (Biorad). Densitometric analysis was performed with Image Lab 5.1 software (Biorad).

### Dot blot analysis

In order to determine the effect of IgG1-iS18 treatment on the insoluble Aβ levels, a dot blot was performed. The GuHCl protein extracts were dotted onto a nitrocellulose membrane and allowed to dry. The membrane was blocked in 3% BSA in PBST for 30 min where after it was incubated with rabbit anti-human Aβ _1-42_ (Cell Signaling Technology^®^, D9A3A) (1:1000) at room temperature for 1 hour. The membrane was washed 3 x with PBST for 5 min and incubated with anti-rabbit HRP secondary antibody (Cell Signalling) (1:2500) at room temperature for 30 min. After the membrane was washed another 3 × with PBST, it was incubated with Clarity™ Western ECL Blotting Substrate (Biorad) and the ChemiDoc™ Imaging System (Biorad) was used for detection. Densitometric analysis was performed with Image Lab 5.1 software (Biorad).

### Aβ_42_ ELISA

Soluble human Aβ_1-42_ was measured with the Quantikine^®^ ELISA Human Amyloid β (aa1-42) kit (R&D Systems). The manufacturer’s instructions were followed. Briefly: The plate was washed twice with Wash Buffer immediately prior to use. Human Amyloid β (aa1-42) standard and 100× diluted soluble protein samples were added to the wells (100 μl) and incubated for 2 hours at 2–8° C. Each well was then aspirated and washed four times with Wash Buffer. Cold Human Amyloid β (aa1-42) conjugate (200 μl) was added to each well and incubated for 2 hours at 2–8° C. The plate was washed four times with Wash Buffer and 200 μl of Substrate Solution was added to each well. The plate was then incubated for 30 min at room temperature in the dark where after 50 μl Stop Solution was added. The optical density was determined using an ELISA plate reader at 450 nm with wavelength correction at 540 nm.

### Telomerase activity

The TRAPeze^®^ Kit, RT Telomerase Detection Kit (Merck), was used to determine the effect of IgG1-iS18 treatment on telomerase activity. Relative telomerase activity was quantified following the manufacturers protocols with minor alterations. Protein and RNA was extracted using 200 μl of CHAPS lysis buffer per 40–100 mg of tissue and incubated on ice for 30 min. In order to obtain the extract, the samples were centrifuged at 16000 × g for 20 minutes at 4° C and the supernatant was snap frozen on dry ice. The protein was quantified with the NanoDrop^®^ ND-1000 (Thermo Scientific) and standardized to 1000 ng/μl for all experimental and control reactions. OneTaq^®^ HotStart Taq Polymerase (5 U/μl) (New England Biolabs) was used and all samples were analysed via qPCR with the Roche LightCycler LC480. The following cycling parameters were applied: 37° C for 30 minutes, 95° C for 2 minutes and 45 cycles of 95° C for 15 seconds, 59° C for 60 seconds and 45° C for 10 seconds. Telomerase activity was calculated from the standard curve generated by 1:10 serial dilutions (20–0.0002 amoles) of TSR8 control template as per Merck Millipore instructions. The data was analysed with LightCycler1 Software version 1.5.1.

### Statistical analysis

Statistical analysis was performed using Microsoft Excel 2010 (Microsoft Corporation) and QuickCalcs Outlier Calculator ©2017 GraphPad Software which employs the Grubbs’ test (extreme studentized deviate). All experiments were performed with a minimum of 6 biological repeats and error bars represent standard deviation. The One-way ANOVA and Student’s *t*-test was performed at a 95% confidence interval; where *p* values < 0.05 were considered statistically significant (^*^p < 0.05, ^**^p < 0.01 and ^***^p < 0.001). The Tukey HSD or Tukey-Kramer Post Hoc Test was performed after One-Way ANOVA to confirm significance.

## SUPPLEMENTARY MATERIALS FIGURES AND TABLE



## Abbreviations

Aβ: Amyloid Beta
AD: Alzheimer’s Disease
APP: Amyloid Precursor Protein
BACE-1: B-site APP cleaving enzyme
BCA: Bicinchoninic Acid Assay
CHAPS: 3-((3-cholamidopropyl)dimethylammonio)-1-propanesulfonate
DDR: DNA Damage Response
DMSO: Dimethyl Sulfoxide
DNA: Deoxyribonucleic Acid
DSBs: Double Stranded Breaks
ELISA: Enzyme-linked Immunosorbent Assay
FAD: Familial Alzheimer’s Disease
GuHCl: Guanidine HCl Buffer
HCl: Hydrochloric Acid
HEK293: Human Embryonic Kidney Cells
HRP: Horseradish Peroxidase
hTERT: Human Telomerase Reverse Transcription
kDa: Kilo Dalton
LRP/LR: 37 kDa Laminin Receptor Precursor/67 kDa High Affinity Laminin Receptor
mTERT: Mouse Telomerase Reverse Transcriptase
PBS: Phosphate Buffered Saline
PBST: Phosphate Buffered Saline with Tween
qPCR: Quantitative Polymerase Chain Reaction
RIPA: Radioimmunoprecipitation Assay Buffer
RNA: Ribonucleic Acid
ROS: Reactive Oxygen Species
shRNA: Short Hairpin RNA
siRNA: Small Interfering RNA
TERC: Telomerase RNA Component
TERT: Telomerase Reverse Transcriptase
