# Supplementary material for: LRP/LR specific antibody IgG1-iS18 impedes neurodegeneration in Alzheimer's disease mice
Source: Oncotarget. 2018 Jun 5;9(43):27059–73. doi: 10.18632/oncotarget.25473 (PMC6007457; doi:10.18632/oncotarget.25473)
Supplement: Supplementary file 1 [file oncotarget-09-27059-s001.pdf]

LRP/LR specific antibody IgG1-iS18 impedes neurodegeneration in Alzheimer's disease mice

SUPPLEMENTARY MATERIALS

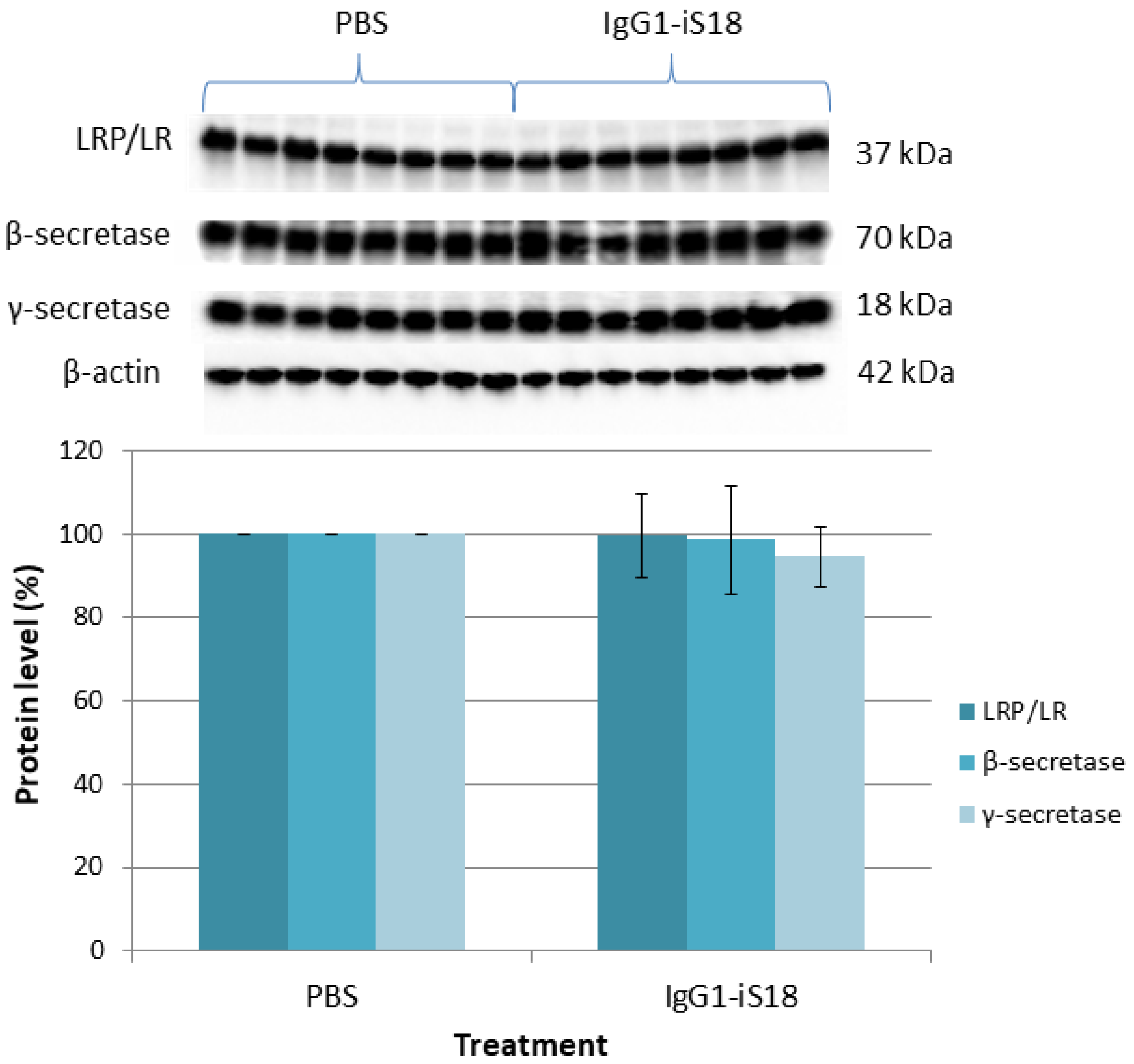

Supplementary Figure 1: Western blot analysis of LRP/LR, β- and γ-secretase protein levels in brain tissue of AD transgenic mice after treatment with IgG1-iS18 and PBS. No significant difference in LRP/LR, β- and γ-secretase protein levels were observed. Error bars represent standard deviation, $n = 8$.

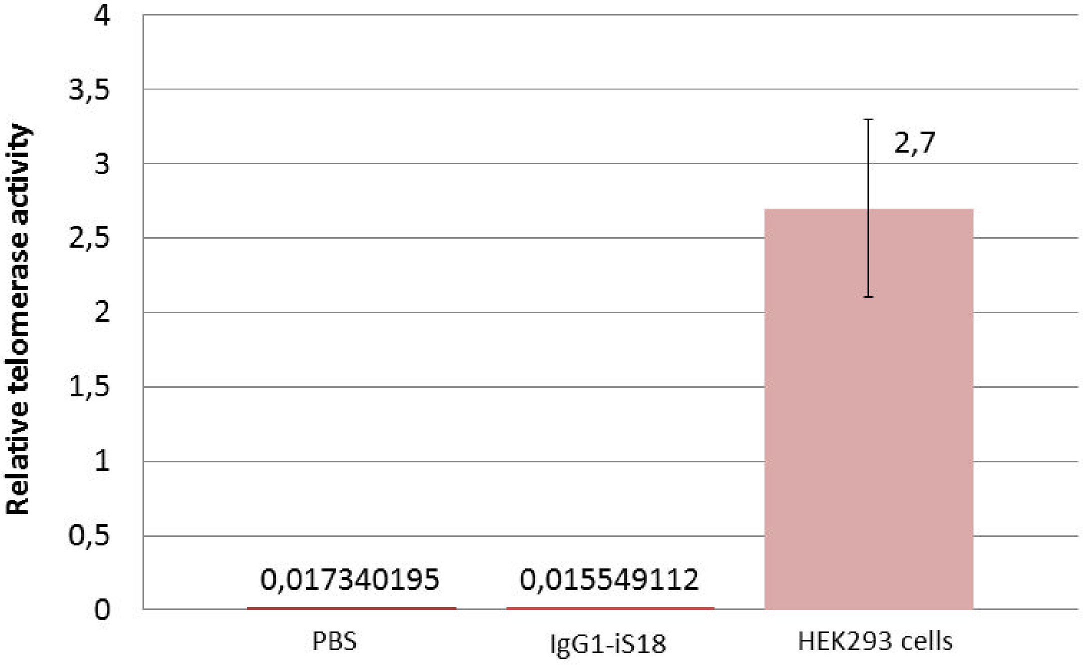

Supplementary Figure 2: Telomerase activity in mouse brain tissue after treatment with PBS and IgG1-iS18. Telomerase activity in the mouse brain tissue was compared to telomerase positive HEK293 cells and is almost negligible compared to the HEK293 control. No significant difference in telomerase activity in brains of AD transgenic mice was observed after treatment with IgG1-iS18. Error bars represent standard deviation, $n = 8$ per treatment group; $n = 6$ for HEK293 samples.

Supplementary Table 1: List of primary and secondary antibodies used for western blot analysis

Target Protein	Primary antibody	Secondary antibody
APP	Rabbit anti-APP,<br>1:1000<br>(abcam 2027)	anti-rabbit IgG-HRP,<br>1:2500<br>(Cell Signaling Technology® 7074S)
mTERT	Rabbit anti-hTERT,<br>1:6666<br>(abcam 183105)	anti-rabbit IgG-HRP,<br>1:2500<br>(Cell Signaling Technology® 7074S)
γH2AX	Rabbit anti-Phospho-γH2AFX (PSER139),<br>1:5000<br>(Sigma SAB4300213)	anti-rabbit IgG-HRP,<br>1:3333<br>(Cell Signaling Technology® 7074S)
H2AX	Rabbit anti- H2A.X (D17A3)<br>1:1000<br>(Cell Signaling Technology® 7631)	anti-rabbit IgG-HRP,<br>1:2500<br>(Cell Signaling Technology® 7074S)
LRP/LR	Human anti-LRP/LR IgG-iS18,<br>1:1000<br>(Affimed)	anti-human IgG-HRP,<br>1:5000<br>(abcam 6858)
β-secretase	Rabbit anti-BACE1,<br>1:1000<br>(abcam 2077)	anti-rabbit IgG-HRP,<br>1:2000<br>(Cell Signaling Technology® 7074S)
γ-secretase	Rabbit anti-PS1,<br>1:1000<br>(abcam 76083)	anti-rabbit IgG-HRP,<br>1:2500<br>(Cell Signaling Technology® 7074S)
β-actin	Murine anti-β-actin-peroxidase,<br>1:3333<br>(Sigma A3854)	-
